# The Implicit Association Test in health professions education: A meta-narrative review

**DOI:** 10.1007/s40037-019-00533-8

**Published:** 2019-09-18

**Authors:** Javeed Sukhera, Michael Wodzinski, Maham Rehman, Cristina M. Gonzalez

**Affiliations:** 1 Schulich School of Medicine and Dentistry, Western University, London, Ontario Canada; 2 Department of Psychiatry, University of Toronto, Toronto, Ontario Canada; 3 Western University, London, Ontario Canada; 4 Albert Einstein College of Medicine/Montefiore Medical Center, New York, USA

**Keywords:** Implicit bias, Implicit Association Test, Reflection, Health disparities

## Abstract

**Introduction:**

Implicit bias is a growing area of interest among educators. Educational strategies used to elicit awareness of implicit biases commonly include the Implicit Association Test (IAT). Although the topic of implicit bias is gaining increased attention, emerging critique of the IAT suggests the need to subject its use to greater theoretical and empirical scrutiny.

**Methods:**

The authors employed a meta-narrative synthesis to review existing research on the use of the IAT in health professions education. Four databases were searched using key terms yielding 1151 titles. After title, abstract and full-text screening, 38 articles were chosen for inclusion. Coding and analysis of articles sought a meaningful synthesis of educational approaches relating to the IAT, and the assumptions and theoretical positions that informed these approaches.

**Results:**

Distinct, yet complementary, meta-narratives were found in the literature. The dominant perspective utilizes the IAT as a metric of implicit bias to evaluate the success of an educational activity. A contrasting narrative describes the IAT as a tool to promote awareness while triggering discussion and reflection.

**Discussion:**

Whether used as a tool to measure bias, raise awareness or trigger reflection, the use of the IAT provokes tension between distinct meta-narratives, posing a challenge to educators. Curriculum designers should consider the premise behind the IAT before using it, and be prepared to address potential reactions from learners such as defensiveness or criticism. Overall, findings suggest that educational approaches regarding implicit bias require critical reflexivity regarding assumptions, values and theoretical positioning related to the IAT.

## What this paper adds?

The Implicit Association Test (IAT) is used in distinct, yet complementary, ways in health professions education. While the dominant perspective utilizes the IAT as a metric of implicit bias to evaluate the success of an educational activity, a contrasting narrative describes the IAT as a tool to promote awareness while triggering discussion and reflection. In this meta-narrative review, the authors found that use of the IAT provokes tension between distinct divergent meta-narratives. Findings suggest that future educational initiatives regarding implicit bias recognition and management may be enhanced by critical reflexivity regarding assumptions, values and theoretical positioning related to the IAT.

## Introduction

Implicit biases include attitudes that form through experiences and operate outside an individual’s awareness. Research on implicit bias in healthcare has found that implicit biases contribute to disparities in cardiac care and pain treatment, among others, adversely influencing several patient populations [1]. Underserved groups often experience bias in healthcare settings that results in inequitable treatment. Such disparities can also exist at the level of the organization or health system, leading to poor outcomes [2]. Implicit biases within health professionals may perpetuate discrimination against patients, even when such professionals are educated about their biases and consciously attempt to suppress them [3]. Social psychology researchers have suggested that implicit biases represent a unique type of prejudice that is inherently complex and includes ambivalent and nuanced attitudes that are more challenging to address than traditional explicit prejudice [4]. Given their unique and complex nature, multiple strategies may mitigate the negative impact of implicit bias. Among those strategies are educational approaches to increase awareness of implicit biases and foster behavioural change among learners [5–8]. A prominent tool used in health professions research to facilitate implicit bias awareness while prompting discussion and reflection is the Implicit Association Test (IAT).

The IAT is an online metric of response time that measures implicit (unconscious) associations between certain concepts [9]. The experience of taking the IAT involves logging into a web-based platform and clicking keys in response to visual representation of specific categories. For example, concepts such as ‘black’ and ‘white’ could be associated with ‘good’ and ‘bad.’ Individual response latency is then calculated and used as a proxy for the strength of implicit associations between categories. Once completing the test, individuals are provided feedback regarding the degree of their associations. For example, they may show a low, moderate or strong association between concepts, or no association at all [9]. The IAT has been researched extensively and found to be insensitive to procedural variation [10] and less susceptible to faking than explicit measures such as questionnaires [11]. The IAT has also demonstrated solid internal consistency, and high test-retest reliability [12–16]. There are multiple versions of the IAT designed to uncover a range of different implicit biases, such as biases relating to age, race, or illness category; however, all operate on the same principles.

Since the introduction of the IAT by social psychology researchers Mahzarin Banaji and Anthony Greenwald in 1998, educational strategies related to implicit bias have varied. Some suggest checklists may be useful while others promote enhancing conscious efforts to overcome biases [17, 18]. Over time, there has been growing recognition that changing biases is difficult because they are reinforced by culture [19, 20]. Therefore, some educators advocate that curricula foster skills to both recognize when bias is activated or perceived in an encounter and mitigate the influence of such bias on outcomes of the encounter [21]. Attention to implicit bias recognition and management also involves potential curricular targets from the patients’ perspective [22]. Educators subscribing to this perspective describe the importance of promoting awareness of one’s implicit biases, utilizing the IAT itself as an educational tool [20]. Together, strategies to promote implicit bias recognition and management appreciate that discussions about implicit bias are unique because they shift the focus of introspection from guilt to responsibility and require a safe learning environment free from blame or criticism to effectively reflect, critically question, and act [20, 23, 24].

Despite its strengths, the IAT is not without criticism. Critics suggest that instead of reflecting authentic negative attitudes, IAT scores may stem from other associations such as victimization, maltreatment and oppression [25–29]. Critiques of the IAT generally address: construct validity (does the IAT truly measure implicit bias?), psychometrics (does the IAT does predict discriminatory behaviour?), and external validity (is the IAT relevant or applicable in real-world contexts?). Critics also suggest that research related to the IAT may be insufficient to advance practical solutions for discrimination and prejudice [29].

In the context of growing interest and controversy regarding implicit bias among health professions educators, we sought to synthesize existing knowledge on how and why the IAT is used for teaching and learning in health professions education. Our focus was broad so that we could capture the traditions, debates, and dilemmas that describe the IAT. We were not seeking a comprehensive summation of all research regarding implicit bias instruction but, rather, a meaningful synthesis of educational approaches relating to the IAT and the assumptions and theoretical positions that inform these approaches. In doing so, we hoped our results could inform future efforts to enhance implicit bias recognition and management within health professions education.

## Methods

### Methodological framework and planning

We chose to conduct a meta-narrative review, which is a method of systematic review that is useful for exploring topics that have been differently conceptualized and studied by different groups of researchers [30]. By its nature, a meta-narrative review captures contradictions within the literature while seeking a meta-narrative to facilitate sense-making of such contradictions [31]. Reviewers follow an iterative process by developing and outlining theoretical frameworks based on individual knowledge and experience on the topic of interest. Next, a focused search is completed to capture and compare salient narratives. These articles are then categorized by trends and compared between the theoretical framings proposed [32, 33]. Consistent with our desire to deepen conceptual and theoretical understanding regarding IAT use, a meta-narrative review provided us with an opportunity for both critical synthesis while capturing and interpreting tensions on how the IAT is used in health professions education. The core principles of meta-narrative review—pragmatism, pluralism, historicity, contestation, reflexivity, and peer review [30]—are reflected throughout our review process.

### Search phase

We began our search by exploring what is known about the use of the IAT in health professions education. More specifically, we sought to understand why and how the IAT was used to promote teaching and learning about implicit bias within adult learners. To illuminate our topic from multiple angles and perspectives, we drew from a broad set of databases including both health professions and non-health professions education literature while only selecting articles that would be relevant to a health professions context. To be included, an article had to describe use of the IAT as part of or related to an educational activity. For example, if an article described the use of the IAT solely to measure attitudes within health professions students, it was not included. We also excluded any dissertations, commentaries, literature searches or articles describing elementary or secondary education.

**Fig. 1:**
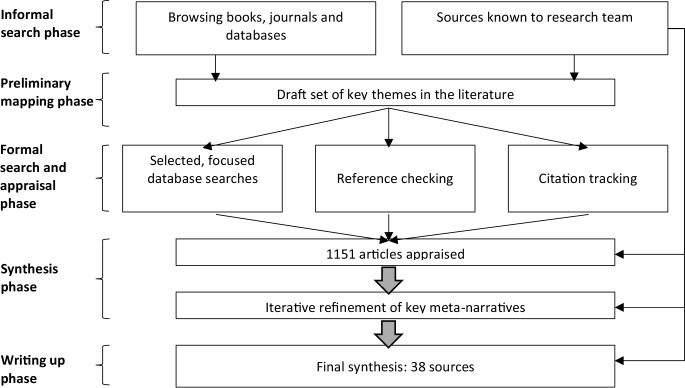
Summary of the phases of the meta-narrative review

In consultation with an academic librarian, four electronic databases (Medline, PsycInfo, ERIC and Web of Science) were searched from May to June 2018 using key search terms. These databases were selected to cover a range of health professions. The search combined the terms (“implicit association test”) AND (educate* OR learn* OR teach*). This initial search was limited to abstracts translated to English and published from 1995 to 29 May 2018. While the IAT was initially published in 1998, its founders first published a paper in 1995 asserting that the idea of implicit bias can apply to social constructs [33]. Our preliminary search yielded 1151 titles. After removing duplicates and conducting title and abstract screening by 2 independent reviewers, 128 full-text articles were reviewed by 2 independent reviewers and screened against our inclusion and exclusion criteria. Any conflicts were resolved through discussion and consensus among all 3 authors. Eventually, 38 articles were chosen for extraction and analysis. Fig. 1 shows a summary of the phases of the meta-narrative review.

### Mapping, synthesis and reflexivity

Our team consisted of the first author (J.S.), a child and adolescent psychiatrist, faculty, and PhD in health professions education, as well as a medical student (M.W.), and undergraduate student in bio-medical sciences (M.R.), and an internist and expert in implicit bias instruction within health professions education (C.G.). While J.S., M.W., and M.R. were involved in extraction and analysis, C.G. offered consultation and input on study design, analysis and manuscript preparation. The first author reviewed each article with 1 other team member reviewing and extracting data. Extraction initially focused on the main goals and aims of research, study design, methods, setting, type of bias (race, age, gender, etc.,) and reviewed how the IAT was used in relation to an educational activity. Since the analysis was an iterative process, the team met at regular intervals to discuss their perspectives on the articles and analyze how findings relate to historical and theoretical approaches on IAT use. The team also checked tracked citations and checked references to trace the epistemic traditions since the initial research on the IAT to build a picture of how research unfolded from this work. In our early analysis, it was clear that the IAT was used either as a metric to measure implicit bias or as a prompt for reflection. We therefore analyzed each article according to ontological and epistemological worldviews. Ontology refers to assumptions about the nature of reality, while epistemology refers to assumptions about the nature of knowledge. For example, was the IAT used to measure the objective reality of an individual’s bias that can be measured, studied and replicated? Or did the use of the IAT reflect multiple possible ways of knowing? [34, 35].

During the search phase, we made the decision to include articles that met our inclusion criteria, however, did not describe IAT use with health professions students. During our analysis we included several articles that used the IAT with undergraduate students, and other populations such as the general public. Therefore, if an article was consistent with our original research question to explore how and why the IAT is used to promote teaching and learning about implicit bias within adult learners, and the use of the IAT was consistent with an educational strategy that could potentially be replicated with health professions students, we included this article for analysis.

## Results

### Main findings: contrasting narratives

Our main findings are summarized in Tab. 1 and 2; the 38 articles selected for inclusion ranged from 2003–2018. Participants included undergraduate students (16 studies), medical students (14 studies), graduate students (2 studies), teacher trainees (2 studies), medical residents (2 studies), occupational therapy students (1 study), dieticians (1 study), social work students (1 study), and the public (1 study).

**Table 1:** Studies which used the Implicit Association Test as a metric of bias

Author/Year	Type of bias	Participant demographic	Educational activity	
*Baker, 2017 *[36]	Weight	Medical students	Medical curriculum	^a^
*Barney, 2017 *[37]	Mental illness	Undergraduate	Service-learning	
*Blincoe, 2009 *[38]	Race	Undergraduate	Cooperative learning	
*Calabrese, 2018 *[39]	HIV/LGBT	Medical students	Case vignette	
*Castillo, 2007 *[40]	Race	Graduate students	Cultural competency training	
*Dobbie, 2015 *[41]	Race	Teacher trainees	Service learning	
*Galli, 2015 *[42]	Disability	Public	Social contact	
*Gonzalez, 2014 *[7]	Race	Medical students	Health disparities session	^b^
*Gutierrez, 2014 *[43]	Race	Graduate students	Videogame	
*Kallman, 2017 *[44]	Disability	Undergraduate	Video	
*Kashihara, 2015 *[45]	Mental illness	Undergraduate	Readings	^c^
*Kogan, 2018 *[46]	Age	Undergraduate	Service learning	
*Lincoln, 2008 *[47]	Mental illness	Undergraduate and medical students	Brochure and video	
*Malinen, 2007 *[48]	Gender	Undergraduate	Multimodal curriculum	
*Meadows, 2017 *[49]	Weight	Medical students	Social contact and empathy training	
*Merz, 2018 *[50]	Age	Undergraduate	Course on aging	
*Omori, 2012 *[51]	Mental illness	Medical residents	Social contact	
*Penner, 2013 *[52]	Race	Family medicine residents	Group intervention	
*Steed, 2010 *[53]	Race	Occupational therapists	Cultural competency workshop	
*Swift, 2013 *[54]	Race	Dieticians and medical students	Video	
*Teachman, 2003 *[55]	Weight	Undergraduate	Reading	
*Wang, 2016 *[56]	Mental illness	Undergraduate and medical students	Social contact	
*Whitford, 2018 *[57]	Race	Pre-service teacher trainees	Empathy training	
*Zvonkovic, 2015 *[58]	Mental illness	Undergraduate	Informative facts	

^a^Used implicit relational assessment procedure not IAT

^b^These authors used the IAT both as a measure, and an elicitation tool

^c^Used brief version of the IAT

**Table 2:** Studies which used the Implicit Association Test to elicit reflection and discussion

Author/Year	Type of bias	Participant demographic	Educational activity	
*Adams, 2008 *[59]	Race	Undergraduate	Tutorial	
*Adams, 2014 *[60]	Race	Undergraduate	Teaching module	
*Casad, 2012 *[61]	Several ^b^	Undergraduate	Essay	^a^
*Gonzalez, 2014 *[7]	Race	Medical students	Health disparities session	^b^
*Gonzalez, 2015 *[21]	Race	Medical students	Health disparities elective	
*Hernandez, 2013 *[62]	Race	Medical students	Group learning	
*Hillard, 2013 *[63]	Race	Undergraduate	Reading material and essay	
*Jarris, 2012 *[64]	Race	Medical students	Lectures and e‑learning	
*Marion, 2018* [65]	Age	Medical students	Multimodal curriculum	
*Morris, 2000* [66]	Race	Undergraduate	Research assignment	
*Nadan, 2017* [67]	Race	Social work students	Reflective essay	
*Siegelman, 2016 *[68]	Race	Emergency medicine residents	Didactic lecture	
*Teal, 2010 *[8]	Race	Medical students	Group discussion	
*VanRyn, 2015 *[69]	Race	Medical students	Medical curriculum	
*Vondras, 2004 *[70]	Age	Undergraduate	E‑learning	

^a^Participants chose any IAT version though 69% took race

^b^These authors used the IAT both as a measure, and an elicitation tool

In general, the IAT was used to measure implicit bias or as a prompt for discussion and reflection. While 24 out of 38 articles used the IAT as a metric of bias [7, 36–58], another 15 articles used the IAT as a stimulus to encourage discussion and reflection [8, 21, 59–71] (one used the IAT for both) [7]. When used as a metric of implicit bias, the IAT was used to calculate the degree of implicit bias, and measure implicit attitudes in relation to an educational intervention. In most of these circumstances, the IAT was given before and after a defined educational intervention. If IAT results revealed a change in implicit bias, the educational intervention was deemed effective by the respective investigative teams.

The IAT was used to measure several different types of bias including biases related to race [38, 40–43, 52–54, 57], age [46, 50], mental illness [37, 45, 47, 51, 56, 58], weight [36, 49, 55], disability [42, 44], gender [48], and others [39]. The IAT was also used to measure the effectiveness of several types of educational activities including experiential learning, didactics, videos, independent-learning, social-contact and small/large group discussion.

Experiential learning involving social contact was a common instructional strategy among medical students and postgraduate medical residents. For example, Meadows analyzed medical student attitudes towards overweight patients before and after contact with overweight individuals [49]. Similarly, Omori used the IAT to measure bias before and after contact with schizophrenia patients, finding some changes in IAT results after their intervention [51]. Out of the articles that used the IAT as a metric and used a pre-post design, only 5 demonstrated a significant change in implicit attitudes after the intervention [38, 40, 46, 49, 57], while 10 clearly demonstrated no significant changes [44, 45, 47, 50–54, 56, 58]. Other papers used the IAT to measure bias in association with educational interventions, however, did not use a pre- and post-design.

In contrast, other authors utilized the IAT as a catalyst for reflection and discussion. In these studies, educational activities included reflective writing, group discussion, and varied regarding the content and type of material used alongside the IAT, frequency and length of the activity. Several studies used the IAT as a demonstration of bias before the start of their learning activity, or as a way of framing implicit bias [60, 62, 67]. Others intended for the IAT to enhance knowledge about implicit bias, improve the design and delivery of their teaching module, or to motivate students to recognize and control their prejudices [61]. Most of these authors coupled the IAT with facilitated discussion, debriefing, and/or reflective narrative writing exercises [64–66, 70]. Overall, there was broad variation regarding the intention behind IAT use and the setting in which it was used.

### Sense-making: is the IAT used in distinct, contrasting, or complementary ways?

Our analysis revealed divergence in perspectives on whether individuals’ biases can be changed, and the extent to which measuring change in bias through the IAT is a meaningful approach to teaching and learning about implicit bias. When used as a metric, the IAT represented a gauge to measure the ‘true’ existence of an individual’s implicit biases. In these studies, implicit bias is viewed as an identifiable phenomenon that is perceived as a barrier to equity. These authors adhere to the positivist epistemological tradition that led to the development of the IAT [71]. They argue that implicit bias is a problem that can be solved through education, and that the IAT is best used to measure an individual’s implicit biases before and after an educational intervention.

In contrast, another group of studies described the IAT was a catalytic reagent. These authors use the IAT to facilitate teaching and learning through implicit bias. When used as a catalyst for reflection, many authors delved deeper into how implicit bias influences an individual’s cognitions, emotions, and behaviour. In these studies, the IAT is designed to help identify strategies to reduce implicit bias [59, 62, 63], while drawing attention to systemic and pervasive injustices faced by marginalized or underserved patient groups. These papers straddled between interpretivist and criticalist worldviews, emphasizing the varying interpretations and implications of implicit bias for the individual learner, while shedding light on broader power relations and perceived injustices within society at large.

While our review of teaching and learning involving the IAT reflects perspectives that may seem epistemologically distinct, our analysis also found a meta-narrative that suggests these worldviews are sometimes epistemologically compatible with one another. In some examples, the use of the IAT as a metric to evaluate the success of a learning activity may complement the view that the IAT facilitates transformative learning through fostering critical consciousness regarding the self, others, and the learning environment [72]. Although we found that intentions and justification for IAT use varied within and across the literature, there were several examples of how measurement of cognitive processes complemented social-constructivist and critical approaches to education. For example, Morris and Ashburn-Nardo’s paper explored the affective impact of receiving feedback from the IAT, while investigating whether taking the IAT makes students aware of the possibility that they might harbour implicit biases. Beliefs regarding the IAT were measured using a 15-point scale, while affect ratings were measured using similar techniques [66]. Similarly, Gonzalez, Kim and Marantz used the IAT to trigger discussion in a 2-hour course and subsequently surveyed students about their IAT results, their attitudes and experiences regarding health equity and the potential impact of their biases [7]. Both papers assumed that implicit bias is an entity that can be measured, yet also used the IAT as an elicitation tool for teaching and learning regarding implicit bias.

## Discussion

Conducting a meta-narrative review of how the IAT is used in health professions education reveals two distinct research traditions which may be considered either contrasting or complementary, posing a challenge to health professions educators. Therefore, our findings suggest that any research involving implicit bias or the IAT in health professions requires proactive critical examination of both ontological and epistemological positioning. An ontological position refers to a researcher’s relationship with what they consider real. When discussing implicit bias, health professions educators must ask whether they believe the IAT is a ‘true’ metric of bias that can be changed through their intervention, or if they view the IAT as a tool to facilitate teaching and learning regarding implicit bias. The suggestion that critical examination is required before using the IAT is not new, and aligns with the views of the IAT’s founders, Banaji and Greenwald, who argue that their work on implicit bias has contributed to a ‘revolution’, which demands researchers’ careful attention to the justification for their work [71]. Research on implicit bias and health professionals recognizes that addressing implicit bias requires accepting that not all biases can be eliminated, and therefore, approaches to cultural competence that perpetuate shame or guilt in learners may have unintended consequences [73]. Our analysis of IAT use reinforces that the IAT may be used in distinct yet complementary ways. We emphasize that regardless of how the IAT is used in education, curriculum designers and educators must consider both the premise behind the test, and potential reactions from learners, and have a plan in place to address such reactions prior to delivering instruction [74, 75].

The articles analyzed in our review also provide insights for understanding and addressing critique regarding the IAT. Several of the studies we reviewed explicitly tackled the issue of defensiveness or criticism regarding the test. For example, Gonzalez, Kim and Marantz used the IAT as a reflective trigger for discussion, surveyed students on their perceptions of the influence of bias on clinical care, their IAT results, and various aspects of healthcare disparities. They compared the results of answers to survey items between two groups of students, ‘acceptors’ and ‘deniers’, of the possibility that implicit bias could influence their clinical practice behaviours and other variables [7]. VonDras and Lor-Vang went further by utilizing the IAT as a tool to increase awareness, while stimulating reflection regarding factors which mediate scepticism towards the IAT [70]. In another study, authors explored the idea that the IAT produces negative emotions and whether debriefing alleviates defensive responses regarding the test [66]. Therefore, if the IAT is framed as a definitive metric of bias, it may be more challenging to constructively explore criticisms regarding the test. However, when used to facilitate ‘consciousness-raising’ of potential hidden acts of discrimination and prejudice, attention to IAT critique may complement the critical reflexivity intrinsically embedded within such approaches [72, 75].

Our decision to include several studies that are not rooted in health professions education also merits further elaboration. We included articles that explored the use of the IAT to foster implicit-bias related instruction. These included articles where the IAT was used with undergraduate students, graduate students, teacher trainees, and the public. The deliberate decision to include articles of ‘relevance’ to a health professions context was made with the objectives of a meta-narrative review in mind. Such reviews consider the research traditions on the topic of the IAT, how each tradition has conceptualized the topic, and what insights can be drawn by comparing findings [30]. During analysis, we found that the IAT was used similarly across and between different participant groups. Therefore, meta-narratives regarding IAT use differed according to similar research traditions in both health professions and non-health professions literature.

While our review was not intended to serve as a scoping review, we were still struck by a gap in research related to the IAT in the field. Most of what is known about the IAT comes from a cross-sectional or time-limited analysis of educational activities. Authors have begun to question the extent to which singular interventions may achieve meaningful outcomes related to implicit biases [76, 77]. Research for using the IAT as a metric or for evaluating the efficacy of a curricular intervention is limited. There is also limited evidence that the goal of bias reduction alone is sustainable. This is important in the context of constraints inherent to any health professions curriculum and prioritizing limited resources and time. Therefore, addressing the adverse impact of implicit bias also requires attention to how implicit bias functions within organizations and institutions, and how sociocultural factors may influence teaching and learning activities about bias. Further, in the context of a debate on whether bias-related education seeks to reduce bias or simply manage it, we also suggest that future research should consider how implicit bias recognition and management curricula not only initiate, but also sustain change.

## Limitations

The results of this meta-narrative analysis must be considered within the context it was conducted. The analyses solely reviewed empirical articles, while excluding forms of scholarly information such as dissertations, non-empirical research, and literature reviews. In addition, only studies of an experimental or evaluative nature were included in the analyses. Consequently, future studies are encouraged to include a more diverse sample of scholarly information to ascertain the role of the IAT in health professions education. In addition, our attempts to seek peer review were not extensive and may have benefitted from broader consultation. Lastly, while the decision to include studies that are not rooted in health professions education literature may be considered a limitation, it was made with the principles of a meta-narrative review in mind.

## Conclusion

Overall, our findings suggest that the very nature of implicit bias is too complex to be reduced to activities and methodologies related to time-limited use of the IAT. While the test itself is a useful prompt, this review emphasizes that the IAT is only one small piece of a larger, interconnected set of components related to the process of recognizing and managing biases. Proponents of the emerging theoretical and methodological paradigm of intersectionality argue that efforts to address overlapping and interdepending systems of discrimination and prejudice must move forward despite their contested and unpredictable nature [78]. Therefore, advancing implicit bias recognition and management cannot stall. Critical reflexivity regarding assumptions, values and epistemological positioning related to the IAT will help extend our thinking about the nature of implicit bias and its implications for education, health and social justice.

## References

[1] Hall WJ, Chapman MV, Lee KM (2015). Implicit racial/ethnic bias among health care professionals and its influence on health care outcomes: a systematic review. Am J Public Health.

[2] Nelson AR, Smedley BD, Stith AY (2003). Unequal treatment: confronting racial and ethnic disparities in health care.

[3] Stone J, Moskowitz GB (2011). Non-conscious bias in medical decision making: what can be done to reduce it?. Med Educ.

[4] Gaertner SL, Dovidio JF (1986). The aversive form of racism.

[5] Byrne A, Tanesini A (2015). Instilling new habits: addressing implicit bias in healthcare professionals. Adv Health Sci Educ Theory Pract.

[6] Carnes M, Devine PG, Baier Manwell L (2015). The effect of an intervention to break the gender bias habit for faculty at one institution: a cluster randomized, controlled trial. Acad Med.

[7] Gonzalez CM, Kim MY, Marantz PR (2014). Implicit bias and its relation to health disparities: a teaching program and survey of medical students. Teach Learn Med.

[8] Teal CR, Shada RE, Gill AC (2010). When best intentions aren’t enough: helping medical students develop strategies for managing bias about patients. J Gen Intern Med.

[9] Greenwald AG, McGhee DE, Schwartz JL (1998). Measuring individual differences in implicit cognition: the implicit association test. J Pers Soc Psychol.

[10] Nosek BA, Greenwald AG, Banaji MR (2005). Understanding and using the Implicit Association Test: II. Method variables and construct validity. Pers Soc Psychol Bull.

[11] Steffens MC (2004). Is the implicit association test immune to faking?. Exp Psychol.

[12] Greenwald AG, Nosek BA (2001). Health of the implicit association test at age 3. Z Exp Psychol.

[13] Greenwald AG, Farnham SD (2000). Using the implicit association test to measure self-esteem and self-concept. J Pers Soc Psychol.

[14] Dasgupta N, McGhee DE, Greenwald AG, Banaji MR (2000). Automatic preference for White Americans: eliminating the familiarity explanation. J Pers Soc Psychol.

[15] Bosson JK, Swann WB, Pennebaker JW (2000). Stalking the perfect measure of implicit self-esteem: the blind men and the elephant revisited?. J Pers Soc Psychol.

[16] Nosek BA, Greenwald AG, Banaji MR, Bargh JA (2007). The implicit association test at age 7: a methodological and conceptual review. Automatic processes in social thinking and behavior.

[17] Burgess D, Van Ryn M, Dovidio J, Saha S (2007). Reducing racial bias among health care providers: lessons from social-cognitive psychology. J Gen Intern Med.

[18] Woolf K, Dacre J (2011). Reducing bias in decision making improves care and influences medical student education. Med Educ.

[19] Hannah SD, Carpenter-Song E (2013). Patrolling your blind spots: introspection and public catharsis in a medical school faculty development course to reduce unconscious bias in medicine. Cult Med Psychiatry.

[20] Sukhera J, Watling C (2018). A framework for integrating implicit bias recognition into health professions education. Acad Med.

[21] Gonzalez CM, Fox AD, Marantz PR (2015). The evolution of an elective in health disparities and advocacy: description of instructional strategies and program evaluation. Acad Med.

[22] Gonzalez CM, Deno ML, Kintzer E, Marantz PR, Lypson ML, McKee MD (2018). Patient perspectives on racial and ethnic implicit bias in clinical encounters: Implications for curriculum development. Patient Educ Couns.

[23] Karani R, Varpio L, May W (2017). Commentary: racism and bias in health professions education: how educators, faculty developers, and researchers can make a difference. Acad Med.

[24] Sharma M (2017). Applying multi-theory model of health behaviour change to address implicit biases in public health. Int J Community Med Public Health.

[25] Uhlmann EL, Brescoll VL, Paluck EL (2006). Are members of low status groups perceived as bad, or badly off? Egalitarian negative associations and automatic prejudice. J Exp Soc Psychol.

[26] Blanton H, Jaccard J, Christie C, Gonzales PM (2007). Plausible assumptions, questionable assumptions and post hoc rationalizations: will the real IAT, please stand up?. J Exp Soc Psychol.

[27] Banaji MR, Nosek BA, Greenwald AG (2004). No place for nostalgia in science: a response to Arkes and Tetlock. Psychol Inq.

[28] Andreychik MR, Gill MJ (2012). Do negative implicit associations indicate negative attitudes? Social explanations moderate whether ostensible ‘negative’ associations are prejudice-based or empathy-based. J Exp Soc Psychol.

[29] Mitchell G, Tetlock PE, Lilienfeld SO, Waldeman ID (2017). Popularity as a poor proxy for utility. Psychological science under scrutiny: recent challenges and proposed solutions.

[30] Wong G, Greenhalgh T, Westhorp G, Buckingham J, Pawson R (2013). RAMESES publication standards: meta-narrative reviews. BMC Med.

[31] Green BN, Johnson CD, Adams A (2006). Writing narrative literature reviews for peer-reviewed journals: secrets of the trade. J Chiropr Med.

[32] Greenhalgh T, Robert G, Macfarlane F, Bate P, Kyriakidou O, Peacock R (2005). Storylines of research in diffusion of innovation: a meta-narrative approach to systematic review. Soc Sci Med.

[33] Greenwald AG, Banaji MR (1995). Implicit social cognition: attitudes, self-esteem, and stereotypes. Psychol Rev.

[34] McMillan W (2015). Theory in healthcare education research: the importance of worldview. Researching medical education.

[35] Kelly M, Ellaway RH, Reid H (2018). Considering axiological integrity: a methodological analysis of qualitative evidence syntheses, and its implications for health professions education. Adv Health Sci Educ Theory Pract.

[36] Baker TK, Smith GS, Jacobs NN (2017). A deeper look at implicit weight bias in medical students. Adv Health Sci Educ Theory Pract.

[37] Barney ST, Corser GC, Strosser GL, Hatch DL, LaFrance K (2017). Service-learning in abnormal psychology: softening the implicit stigma against the mentally ill. Scholarsh Teach Learn Psychol.

[38] Blincoe S, Harris MJ (2009). Prejudice reduction in white students: comparing three conceptual approaches. J Divers High Educ.

[39] Calabrese SK, Earnshaw VA, Krakower DS (2018). A closer look at racism and heterosexism in medical students’ clinical decision-making related to HIV pre-exposure prophylaxis (PrEP): implications for PrEP education. AIDS Behav.

[40] Castillo LG, Brossart DF, Reyes CJ, Conoley CW, Phoummarath MJ (2007). The influence of multicultural training on perceived multicultural counseling competencies and implicit racial prejudice. J Multicult Couns Devel.

[41] Dobbie W, Fryer RG (2015). The impact of voluntary youth service on future outcomes: evidence from Teach For America. B. E. J Econ Anal Policy.

[42] Galli G, Lenggenhager B, Scivoletto G, Molinari M, Pazzaglia M (2015). Don’t look at my wheelchair! The plasticity of longlasting prejudice. Med Educ.

[43] Gutierrez B, Kaatz A, Chu S, Ramirez D, Samson-Samuel C, Carnes M (2014). ‘Fair Play’: a videogame designed to address implicit race bias through active perspective taking. Games Health J.

[44] Kallman D (2017). Integrating disability: boomerang effects when using positive media exemplars to reduce disability prejudice. Int J Disabil Hum Dev Educ.

[45] Kashihara J (2015). Examination of stigmatizing beliefs about depression and stigma-reduction effects of education by using implicit measures. Psychol Rep.

[46] Kogan LR, Schoenfeld-Tacher RM (2018). Participation in an intergenerational service learning course and implicit biases. Educ Gerontol.

[47] Lincoln TM, Arens E, Berger C, Rief W (2008). Can antistigma campaigns be improved? A test of the impact of biogenetic vs psychosocial causal explanations on implicit and explicit attitudes to schizophrenia. Schizophr Bull.

[48] Malinen S, Johnston L (2007). The influence of an equity statement on perceivers’ implicit and explicit associations between males and science. N Z J Psychol.

[49] Meadows A, Higgs S, Burke SE, Dovidio JF, van Ryn M, Phelan SM (2017). Social dominance orientation, dispositional empathy, and need for cognitive closure moderate the impact of empathy-skills training, but not patient contact, on medical students’ negative attitudes towards higher-weight patients. Front Psychol.

[50] Merz CC, Stark SL, Morrow-Howell NL, Carpenter BC (2018). When I’m 64: effects of an interdisciplinary gerontology course on first-year undergraduates’ perceptions of aging. Gerontol Geriatr Educ.

[51] Omori A, Tateno A, Ideno T (2012). Influence of contact with schizoprenia on implicit attitudes towards schizophrenia patients held by clinical residents. BMC Psychiatry.

[52] Penner LA, Gaertner S, Dovidio JF (2013). A social psychological approach to improving the outcomes of racially discordant medical interactions. J Gen Intern Med.

[53] Steed R (2010). Attitudes and beliefs of occupational therapists participating in a cultural competency workshop. Occup Ther Int.

[54] Swift JA, Tischler V, Markham S (2013). Are anti-stigma films a useful strategy for reducing weight bias among trainee healthcare professionals? Results of a pilot randomized control trial. Obes Facts.

[55] Teachman BA, Gapinski KD, Brownell KD, Rawlins M, Jeyaram S (2003). Demonstrations of implicit anti-fat bias: the impact of providing causal information and evoking empathy. Health Psychol.

[56] Wang PW, Ko CH, Chen CS (2016). Changes of explicit and implicit stigma in medical students during psychiatric clerkship. Acad Psychiatr.

[57] Whitford DK, Emerson AM (2019). Empathy intervention to reduce implicit bias in pre-service teachers. Psychol Rep.

[58] Zvonkovic A, Lucas-Thompson RG (2015). Refuting the myth of the ‘violent schizophrenic’: assessing an educational intervention to reduce schizophrenia stigmatization using self-report and an implicit association test. Soc Work Ment Health.

[59] Adams G, Edkins V, Lacka D, Pickett KM, Cheryan S (2008). Teaching about racism: pernicious implications of the standard portrayal. Basic Appl Soc Psych.

[60] Adams VH, Devos T, Rivera LM, Smith H, Vega LA (2014). Teaching about implicit prejudices and stereotypes: a pedagogical demonstration. Teach Psychol.

[61] Casad BJ, Flores AJ, Didway JD (2012). Using the implicit association test as an unconsciousness raising tool in psychology. Teach Psychol.

[62] Hernandez RA, Haidet P, Gill AC, Teal CR (2013). Fostering students’ reflection about bias in healthcare: cognitive dissonance and the role of personal and normative standards. Med Teach.

[63] Hillard AL, Ryan CS, Gervais SJ (2013). Reactions to the implicit associations test as an educational tool: a mixed methods study. Soc Psychol Educ.

[64] Jarris YS, Bartleman A, Hall EC, Lopez L (2012). A preclinical medical student curriculum to introduce health disparities and cultivate culturally responsive care. J Natl Med Assoc.

[65] Marion GS, Hairston JM, Davis SW, Kirk JK (2018). Using standardized patient assessments to evaluate a health literacy curriculum. Fam Med.

[66] Morris KA, Ashburn-Nardo L (2000). The implicit association test as a class assignment: student affective and attitudinal reactions. Teach Psychol.

[67] Nadan Y, Stark M (2017). The pedagogy of discomfort: enhancing reflectivity on stereotypes and bias. Br J Soc Work.

[68] Siegelman JN, Woods C, Salhi B, Heron S (2016). Health care disparities education using the implicit association test. Med Educ.

[69] van Ryn M, Hardeman R, Phelan SM (2015). Medical school experiences associated with change in implicit racial bias among 3547 students: a medical student CHANGES study report. J Gen Intern Med.

[70] VonDras DD, Lor-Vang MN (2004). Using an internet activity to enhance students’ awareness of age bias in social perceptions. Educ Gerontol.

[71] Greenwald AG, Banaji MR (2017). The implicit revolution: reconceiving the relation between conscious and unconscious. Am Psychol.

[72] Sukhera J, Milne A, Teunissen PW, Lingard L, Watling C (2018). Adaptive reinventing: implicit bias and the co-construction of social change. Adv Health Sci Educ Theory Pract.

[73] Sukhera J, Milne A, Teunissen PW, Lingard L, Watling C (2018). The actual versus idealized self: exploring responses to feedback about implicit bias in health professionals. Acad Med.

[74] Sukhera J, Wodzinski M, Teunissen PW, Lingard L, Watling C (2018). Striving while accepting: Exploring the relationship between identity and implicit bias recognition and management. Acad Med.

[75] Gonzalez CM, Garba RJ, Liguori A, Marantz PR, McKee MD, Lypson ML (2018). How to make or break implicit bias instruction: implications for curriculum development. Acad Med.

[76] Jost JT, Rudman LA, Blair IV (2009). The existence of implicit bias is beyond reasonable doubt: a refutation of ideological and methodological objections and executive summary of ten studies that no manager should ignore. Res Organ Behav.

[77] Maina IW, Belton TD, Ginzberg S, Singh A, Johnson TJ (2018). A decade of studying implicit racial/ethnic bias in healthcare providers using the implicit association test. Soc Sci Med.

[78] Chu S, Williams-Crenshaw K, McCall L (2013). Towards a field of intersectionality studies: theory, applications, and praxis. Signs (Chic).

